# Understanding Bacterial Antiviral Defence Systems and Phage Receptors to Better Inform Rational Phage Cocktail Design to Treat Bacterial Canker

**DOI:** 10.1111/1751-7915.70232

**Published:** 2025-09-19

**Authors:** Kieran Cooney‐Nutley, Sneha Chakravorty, Imogen Nix, Ziyue Zeng, Shannon F. Greer, Mojgan Rabiey

**Affiliations:** ^1^ School of Life Sciences Gibbet Hill Campus, University of Warwick Coventry UK; ^2^ School of Biosciences University of Birmingham Birmingham UK

**Keywords:** antiviral defence system, bacteriophage, host range, lipopolysaccharide, *Prunus avium*, *Pseudomonas syringae*

## Abstract

*Pseudomonas syringae* is a plant pathogen complex responsible for bacterial canker in cherry. In the absence of any control measures, bacteriophages (phages) have the potential for biocontrol. However, it is crucial to first evaluate the role of bacterial antiviral defence systems (ADS) in phage infection dynamics for careful design of a phage cocktail (mixture). Investigating 250 *Pseudomonas* strains revealed the *Ps* complex possessed diverse ADS with defence profiles being influenced by phylogeny. Phage host range assays revealed five MR phages with distinct genotypes possessed strong lytic activity against several bacterial canker‐causing *Ps* pathovars, including *syringae* and *morsprunorum* race 1 and 2. Phage susceptibility and resistance appeared to be associated with individual ADS rather than defence profiles as a whole. Multisequence alignment of lipopolysaccharide biosynthesis genes glucose‐1‐phosphate thymidylyltransferase (*gpt*), glycosyltransferase family 1 (*gst1*) and lipopolysaccharide kinase (*lpk*) found these potential receptor genes to be highly conserved within *Ps* phylogroups. However, *gpt* alone appeared to influence phage infectivity. Our findings indicate that the *gpt* gene is a potential primary predictor of MR phage susceptibility, hypothesised to influence phage absorption, while individual ADS only have a secondary role in phage resistance. This study highlights that understanding the genetic mechanisms underlying phage‐bacterial interactions is crucial for designing more effective phage cocktails capable of targeting a broader range of pathogenic strains, but phage screening still is a powerful tool to select phages for biocontrol treatments.

## Graphical abstract

Correlation between phage host range, antiviral defence systems, and phage receptors revealed the importance of genetic mechanisms underlying phage‐bacterial interactions in designing more effective phage cocktails capable of targeting a broader range of pathogenic strains. However, phage screening still is a powerful tool to select phages for biocontrol treatments.

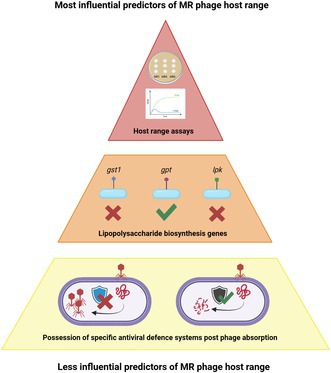

## 1 Introduction

Sweet cherries ( *Prunus avium* L.) are an important crop with an estimated 3 billion tonnes harvested globally each year (FAOSTAT 2024). In the UK, there are approximately 759 ha of commercial cherry orchards that produce £24 million worth of cherries and £3.2 million in exports annually (FAOSTAT 2024; HMRC 2025). *Pseudomonas syringae* (*Ps*) is a bacterial species complex responsible for bacterial canker in *Prunus* spp. and in over 180 other plant species (Crosse 1966), like apple, barley, hazelnut, kiwi, tobacco, tomato and wheat (Bradbury 1986; Janse et al. 1996; Wenneker et al. 2012; Vanneste et al. 2014). *Ps* infection has been particularly devastating to young cherry orchards, with reported losses of up to 75% (Spotts et al. 2010). Disease symptoms can be diverse because pathovars within the *Ps* complex occupy many different niches on and within the cherry tree. Symptoms include fruit spot, shoot necrosis, blossom blight, leaf ‘shot hole’, necrotic cankers, branch dieback and gummosis (Hulin et al. 2020), which impact overall crop yield and quality.

The *Ps* complex is classified into 13 distinct phylogroups, 23 separate clades and 60 pathovars identified to date (Berge et al. 2014; Hulin et al. 2020). Of these pathovars, four have been associated with bacterial canker in cherry. These include *Ps* pv. *syringae* (*Pss*), *morsprunorum* races 1 and 2 (*Psm* R1 and *Psm* R2), *avii* and *cerasi*. *Pss* is a highly diverse clade which includes strains of variable host specificity able to infect different *Prunus* spp., with many strains possessing the ability to cross‐infect (Hulin, Mansfield, et al. 2018). *Pss* strains originate from phylogroup 2 and have diverse lineages descended from distinct sub‐phylogroups, 2b or 2d (Hulin, Armitage, et al. 2018). Closely related *Pss* strains can possess dissimilar host ranges and symptomatology. *Psm* R1 and R2 originate within phylogroups 3 and 1b respectively and have both evolved within clades capable of infecting woody tissue (Berge et al. 2014). Both *Psm* races are limited in host range; *Psm* R1 is specific to cherry and plum, while *Psm* R2 strains only infect cherry. Despite their designations suggesting a close relationship, *Psm* R1 and R2 have distant phylogenetic relations within the *Ps* complex (Berge et al. 2014; Hulin, Armitage, et al. 2018). *P. cerasi* and *Ps* pv. *avii* are less predominant canker‐producing pathogens in cherry and are located within phylogroups 2a and 1a respectively (Ahmadi et al. 2018; Hulin, Mansfield, et al. 2018).

The genetic diversity between *Ps* clades makes breeding for varietal resistance challenging. Furthermore, the exchange of copper and antibiotic resistance genes between clades via horizontal gene transfer reduces the efficiency of these traditional control options. Bacteriophages (phages), viruses that infect and kill bacteria, are an alternative biocontrol method for bacterial canker. The ubiquity of phages, their ability to persist in the environment, their lytic life cycle and their highly specific nature make them a promising new candidate for bacterial disease control not only in agriculture but also in livestock and in humans. Previous work by Rabiey et al. (2020) identified several phages (named MR phages) capable of infecting and lysing *Pss*, *Psm* R1 and *Psm* R2 bacterial isolates but unable to infect the beneficial *Pseudomonas fluorescens* , which helps suppress pathogen growth while also enhancing nutrient availability in plants (Rabiey et al. 2020). Genome sequencing of MR phages revealed five distinct phage genotypes within this group. These phages reduced the bacterial population significantly in vitro and *in planta* (Rabiey et al. 2020).

Bacteria exist within an environment under constant challenge from mobile genetic elements, such as phages, and consequently there exists a high selection pressure for strategies which protect against phage infection. These strategies can target the different stages of the phage infection cycle and can broadly be summarised as prevention of initial phage absorption; prevention of viral DNA injection into the bacterial cytoplasm; targeting, cleavage and exclusion of viral DNA; and prevention of phage replication (Azam and Tanji 2019). Modification to the bacterial cell surface by mutation, masking, downregulation or complete removal of phage receptors reduces phage absorption and is a commonly observed pathway to phage resistance (Manning and Reeves 1978; León and Bastías 2015; Holguín et al. 2019; Koderi Valappil et al. 2021). Receptor mutations can appear rapidly and proliferate quickly throughout bacterial communities if they are advantageous, for example, confer resistance to phage. Therefore, when selecting phages for a cocktail, it is important to consider those with diverse receptor targets to help safeguard against the development of phage resistance. Additionally, receptor density has been demonstrated to result in increased phage absorption; therefore, it stands to reason that a wider range of target receptors would likely also increase phage absorption into the cell as there would be more available, unsaturated target sites (Schwartz 1976). For example, Rabiey et al. (2024) demonstrated that lipopolysaccharide (LPS)‐associated genes serve as the primary receptor for all five MR phages, as deletion of genes involved in LPS synthesis significantly affected phage infectivity, indicating that these phages rely on LPS for binding to and infecting *Pss*.

Antiviral defence systems (ADS) are found within all prokaryotic genomes and have evolved over the millennia to defend against infection by viral DNA. Numerous ADS have been identified to date and are catalogued on open‐access web servers, such as DefenseFinder (https://defensefinder.mdmlab.fr) and PADLOC (https://padloc.otago.ac.nz/padloc/). ADS are generally classified into three main groups: systems which degrade viral DNA; those which inhibit viral DNA or RNA synthesis; and abortive infection (Abi) systems which initiate cell death (Lopatina et al. 2020; Tesson et al. 2022). Several Abi systems have been identified to date (e.g., CBASS, Gabija, Lamassu‐Fam), however, the molecular mechanism by which many of these systems work is yet to be elucidated. Generally, Abi systems work by targeting the cellular machinery required by the phage to replicate. While this arrests phage replication, the inhibition or impairment of vital cellular machinery ultimately results in cell death. This altruistic cellular suicide benefits the surrounding microbial community by curtailing further viral proliferation and infection. Restriction modification (RM) systems work by differentiating ‘self’ prokaryotic DNA and ‘non‐self’ viral DNA and are classified into four system types (RM I to IV), which are further divided into several subtypes. Many ADS have been identified through a ‘guilt‐by‐association’ hypothesis; their proximity within the prokaryotic genome to or genetic homology with established ADS has only suggested their role in defence (Doron et al. 2018). RloC is one such system, its function remains unknown, yet its homology with the previously defined PrrC system suggests it likely functions as an anticodon nuclease in an ADS (Davidov and Kaufmann 2008).

The efficacy of multi‐receptor phage cocktails has been thoroughly investigated (Tanji et al. 2004; Altamirano and Barr 2015; Merabishvili et al. 2017; Li et al. 2023), however, only a few studies have evaluated how the presence of ADS may impact phage infectivity. In this study, we use strains from the *Ps* complex to evaluate how bacterial ADS and receptor variation influence phage host range, which ultimately will help guide future rational phage cocktail design. Such knowledge is critical for optimising phage‐based biocontrol strategies in agriculture and could serve as a framework for more targeted applications in clinical settings, where tailoring phage combinations to the specific defence and receptor landscape of pathogenic strains can improve therapeutic efficacy.

## 2 Materials and Methods

### 2.1 Bacteria Strains

*Ps* strains (listed in Table S1) were obtained from the National Institute of Agricultural Botany—East Malling Research and University of Warwick Stratford Innovation Campus. The strains were grown on King's B (KB) agar plates at 27°C or in liquid culture at 27°C, 200 rpm (King et al. 1954). Frozen stocks were made by incubating each colony in KB for 24 h and storing in an equal volume of 40% glycerol at −80°C.

Genomes of 250 *Pseudomonas* strains, 177 of which were *Ps* strains, were obtained from the NCBI database. Three lipopolysaccharide (LPS) biosynthesis genes, including *gst1* (glycosyltransferase family 1, BKC06_002880), *lpk* (lipopolysaccharide kinase, BKC06_002845) and *gpt* (glucose‐1‐phosphate thymidylyltransferase, BKC06_005130) from the strain syr9097 (accession number: CP026568), identified as MR receptors in Rabiey et al. (2024), were used as references to retrieve homologues from other *Pseudomonas* genomes using the NCBI BLAST discontiguous megablast alignment tool (Camacho et al. 2009).

### 2.2 Bacteriophages

Phages were previously isolated from soil samples of diseased or healthy cherry trees collected at cherry orchards in the Southeast UK by Rabiey et al. (2020). Five phages were used, named MR1, MR4, MR6, MR14 and MR15. Phages MR1, MR4, and MR6 belonged to the family *Podoviridae*, MR14 to *Myoviridae*, and MR15 to *Siphoviridae*. Phages were amplified by adding 100 μL of phage at approximately 10^6^ plaque‐forming units (pfu) mL^−1^ and 100 μL of the host strain syr9097 adjusted to OD_600_ = 0.2 to 5 mL soft KB agar (0.75% agar), which was subsequently used to overlay a standard KB agar plate (1.5% agar). Following overnight incubation at 27°C, 10 mL of phosphate buffered saline (PBS) was added to each plate and left to incubate at room temperature for 15 min with agitation every 5 min. PBS was drawn up from each plate and filtered through a 0.22 μm sterile PES filter.

Spot assays were performed to check titres of phage stocks. 3 μL serial dilutions of phage (10^−1^ to 10^−12^ in PBS) were spotted in duplicate onto KB agar plates overlaid with 5 mL soft agar containing 100 μL of syr9097 overnight culture at OD_600_ = 0.2 and incubated at 27°C overnight. Plaques formed on these plates were used to calculate phage titre.

### 2.3 Host Range Assays

Killing curve assays were performed to assess the host range of the five MR phages against 76 *Ps* strains. Using a Greiner 96‐well flat bottom microplate, 100 μL of *Ps* culture (OD_600_ = 0.2, approximately 2 × 10^8^ cfu mL^−1^) was pipetted in duplicate. 100 μL of phage suspension was added to one well and 100 μL of sterile PBS was added to the adjacent well as a negative control. For each plate and phage, syr9097 was included to confirm the vitality of the phage used and to act as a point of comparison. Blank controls of 200 μL sterile KB broth and 200 μL sterile PBS were included on each plate. Optical density was measured using the Spark Multimode Microplate Reader (Tecan, Männedorf, Switzerland) at 600 nm every 20 min for 24 h, with each reading preceded by a 10 s shaking interval. Plates were incubated at 27°C for the entire duration of the assay. The assay was repeated for each strain for each phage in triplicate. Phage infectivity was categorised as: fully lysed (complete absence of bacterial growth), no lysis (full bacterial growth) and semi lysed (reduced bacterial growth compared to the control with full growth).

### 2.4 Antiviral Defence System Analysis

FASTA files of the 250 *Pseudomonas* genomes were uploaded to the DefenseFinder web service (https://defensefinder.mdmlab.fr/) to identify ADS in each bacterial genome (Tesson et al. 2022). Results were visualised and tested against factors for significance.

### 2.5 Data Analysis

Figure generation and data visualisation were performed on the RStudio software (version 2024.12.1+563) using the packages ComplexHeatmap (version 3.21), pheatmap (version 1.0.12) and ggplot (version 3.3.5) (Wickham and Sievert 2009; Gu et al. 2016; Kolde 2019). Phylogenetic trees were assembled using the Interactive Tree of Life tool, after multi‐sequence alignment using EMBL‐EBI's Clustal Omega (Sievers et al. 2011; Letunic and Bork 2021). Univariate general linear models were used to assess which factors may influence ADS count, at a significance level of 0.01. Pearson correlation was used to assess the relationship between phage susceptibility and presence of different ADS types at significance levels of 0.001, 0.01 and 0.05. Fisher's exact test was used to perform pairwise analysis of ADS types at a significance level of 0.05 and then these values were used to calculate odds ratios (OD). Statistical analysis was performed using the IBM SPSS Statistics version 30 and RStudio software (version 2024.12.1+563).

## 3 Results

### 3.1 The Host Range of MR Phages Does Not Correlate With Host Factors

**FIGURE 1:**
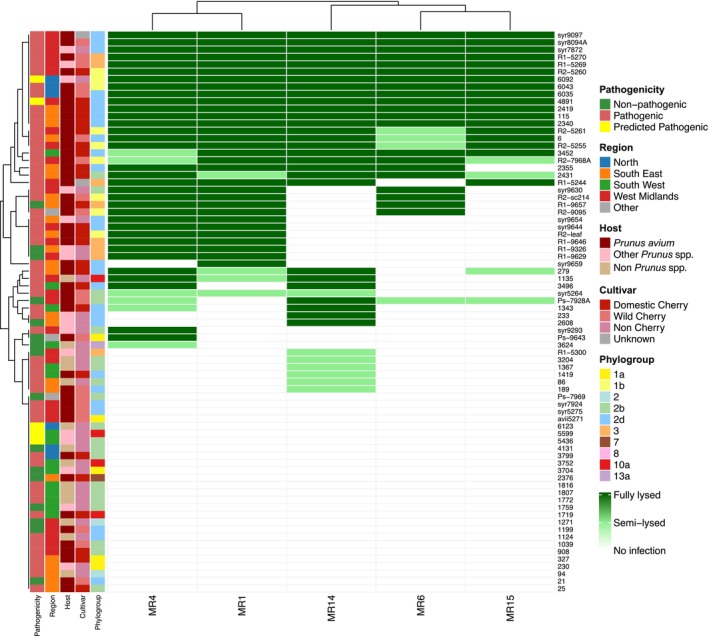
Host range of five MR phages across 76 *Ps* strains. Dark green indicated that strains were strongly lysed by phages, light green indicated that they were weakly lysed and white showed they were resistant to lysis or infection. Strain names were accompanied by metadata concerning their pathogenicity to cherry, region of isolation, the plant species and cultivar they were originally isolated from and phylogroup within the *Ps* complex. Phages were clustered by host range and *Ps* strains were clustered by their phage susceptibilities.

Killing curve assays were used to evaluate the host range of five MR phages and determine whether host factors, such as pathogenicity, region, plant host/cultivar, or phylogroup, influence phage infectivity (Figure 1). Seventy‐six *Ps* strains were selected based on prior characterisation (such as pathogenicity on cherry), and phages representing five genotypes with known lytic activity against *Pss, Psm* R1 and *Psm* R2 were tested (Hulin, Armitage, et al. 2018; Hulin, Mansfield, et al. 2018; Rabiey et al. 2020). Phages were grouped into two host range clusters: MR1 and MR4 formed one group, while MR6, MR14, and MR15 formed the other. MR4 had the broadest host range, fully lysing 34 strains and semi‐lysing 6. MR15 had the narrowest, infecting only 22 strains, with full lysis in 18. Bacterial strains clustered into three categories: highly susceptible, moderately susceptible, and resistant. Several strains showed unique susceptibility; for example, strains #86, #189 and #233 were only lysed by MR14; #3624 and syr9293 by MR4; and #1343 by both. Twelve strains, including #115, #2340, #2419, #4891, #6035, #6043, #6092, R1‐5269, R1‐5270, R2‐5260, syr7872 and syr8094A, shared the same broad susceptibility as control strain syr9097, being fully lysed by all five phages. In contrast, 35.5% of strains were resistant to all five phages, and 15.8% were susceptible to only one. No clustering of susceptibility patterns was observed based on pathogenicity, region, phylogroup, host species, or cultivar, suggesting these factors do not influence phage host range or the infectivity of individual phages.

### 3.2 Pseudomonads Possess Diverse Antiviral Defence Systems With Phylogentically Related Strains Sharing Similar Defence Profiles

**FIGURE 2:**
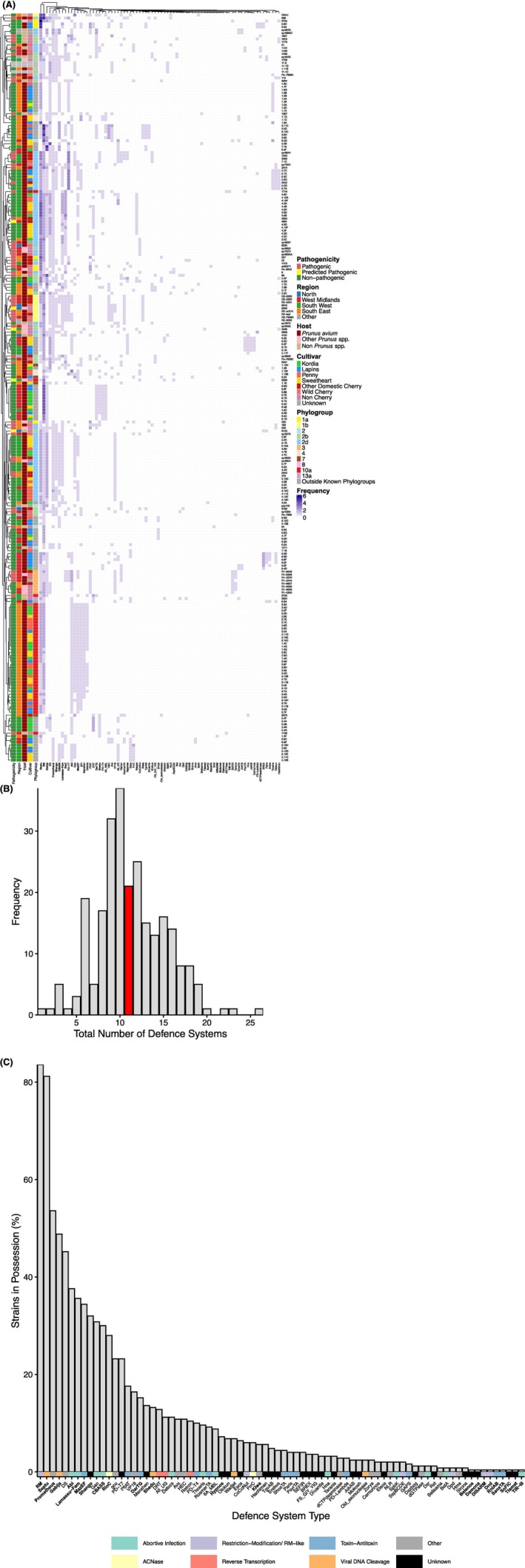
Antiviral defence system (ADS) analysis of 250 *Pseudomonas* strains, including strains from the *Ps* complex. (A) Presence (purple) and absences (white) of different ADS types in *Pseudomonas* stains. Darker shades of purple indicate that multiple copies of the defence system gene were present, or multiple subtypes of said system were present. Strain names were accompanied by metadata concerning their pathogenicity to cherry, region of isolation, the plant species and cultivar they were originally isolated from and phylogroup within the *Ps* complex. (B) Frequency of total number of ADS identified per *Pseudomonas* strain. The median total number has been highlighted in red. (C) Prevalence of different ADS types in *Pseudomonas* strains. ADS types are colour coded by mechanism in which they protect the bacterial cell, as stated on the DefenseFinder webservice wiki (Tesson et al. 2022). ADS types for which the mechanism remains unknown at the time of writing are indicated in dark grey. *Ps* strains were clustered by their ADS profile. ADS discussed within the text are in bold.

ADS were predicted in 250 *Pseudomonas* genomes (including those 76 used in the host range assays) to assess their diversity and identify host factors shaping defence profiles. In total, 132 unique ADS types and 179 subtypes were identified. ADS types represent broad defence mechanisms (e.g., Abi, RM), while subtypes reflect molecular variants of these systems with distinct targeting mechanisms. *Ps* strains carried diverse combinations of ADS types (Figure 2A), with strains clustering by phylogroup based on their defence profiles. For instance, *Psm* R1 (phylogroup 3) and *Psm* R2 (phylogroup 1b) formed distinct clusters. Strains in phylogroups 1a and 1b grouped by sub‐phylogroup, showing distinct defence signatures, while phylogroup 2 strains clustered together regardless of sub‐phylogroup (2b or 2d), though they formed three distinct defence clusters. Strains outside defined phylogroups also clustered phylogenetically. Factors such as pathogenicity, region, host, or cultivar showed no association with defence profiles. The number of ADS per strain varied considerably (Figure 2B), with strain #1271 possessing the fewest and strain #908 the most. RM systems were the most common, found in 83.2% of strains, followed by Septu, Abi, Prometheus, Gabija, Lamassu‐Fam, CBASS, RloC, Shango and MazEF (Figure 2C). Septu was the most abundant ADS overall, with 392 instances across 80.4% of strains. Of these, 45.8% had two copies, 12.9% had three and 7% had four or more, suggesting either functional redundancy or unclassified subtypes not distinguished by DefenseFinder. Few ADS types only appeared once, such as Belenos, DISARM, SoFIC, RnlAB, DnD, Damona, TIR‐III, Tiamat, SanaTA. Strains lacking RM or Septu were almost exclusively from outside the *Ps* complex. Phylogroup was significantly associated with ADS abundance (*p* < 0.01; Table S2).

**FIGURE 3:**
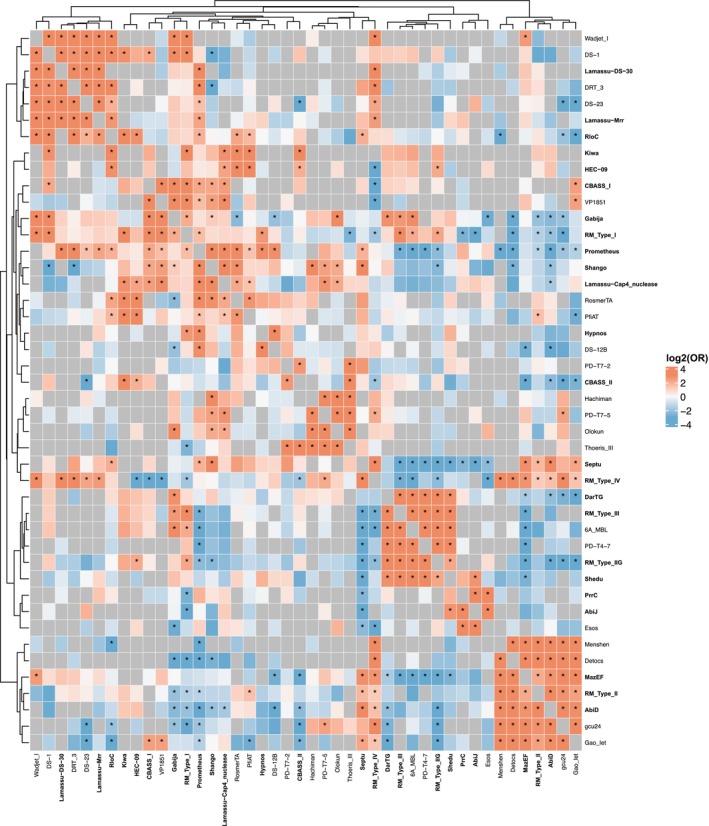
Co‐occurrence of antiviral defence system (ADS) subtypes in 250 *Pseudomonas* strains. Only ADS subtypes present in 10% of the strains were selected for analysis. Fisher's exact test identified significant associations between system subtypes (*p* < 0.01, *) and was used to calculate the log of the odds ratio (log [OR]). Red shades indicate a positive log [OR] and positive co‐occurrence of subtype genes, while blue shades indicate a negative log [OR] and negative co‐occurrence of subtype genes. Null values, caused by self‐association or zero variation, are shown in grey. ADS discussed within the text are in bold.

Many ADS types were further distinguished into subtypes. RM Type I was the most common RM subtype identified, followed by Type IV, Type II, Type III and Type IIG respectively. Diverse Abi (AbiD, AbiJ, AbiAlpha, AbiH, AbiU, AbiV, AbiE, AbiL, AbiO), Lamassu‐Fam (Lamassu‐Cap4_nuclease, Lamassu‐Mrr, Lamassu‐DS‐30, Lamassu‐Hydrolase_Protease) and CBASS (CBASS_II, CBASS_I, CBASS_III) subsystems were also identified. ADS subtypes showed distinct patterns of co‐occurrence (Figure 3). RM Type I was significantly more likely to co‐occur with Type IIG or III, but less likely with Type II or IV (*p* < 0.01). Conversely, Type IV was more likely to occur with Type II, but rarely with Type I, IIG or III (*p* < 0.01). This suggests mutual exclusivity among some subtypes, likely due to functional redundancy and fitness costs. Maintaining multiple similar systems may offer no added benefit, while co‐occurring subtypes may complement each other by covering different defence gaps or ‘blind spots’.

Analysis of the correlation between ADS and phage susceptibility revealed that some closely related ADS exhibited opposite associations with phage susceptibility. PrrC, a homologue of RloC, is linked to resistance to MR1 and MR4 (*p* < 0.05). Most strains with PrrC also carried RloC but were resistant to all five phages, unlike strains with RloC alone, which were susceptible. RM Type II is associated with susceptibility to MR1, MR6, MR15 (*p* < 0.01), and MR4 (*p* < 0.05), while RM Type IIG is linked to resistance to MR1, MR4, and MR14 (*p* < 0.05). Similarly, Lamassu‐DS‐30 is associated with susceptibility to MR1 and MR6 (*p* < 0.001), and MR4, MR14, MR15 (*p* < 0.01), whereas Lamassu‐Mrr is associated with resistance, though not significantly.

### 3.3 Small Differences in Bacterial Profiles Can Result in Resistance to Phage Lysis

**FIGURE 4:**
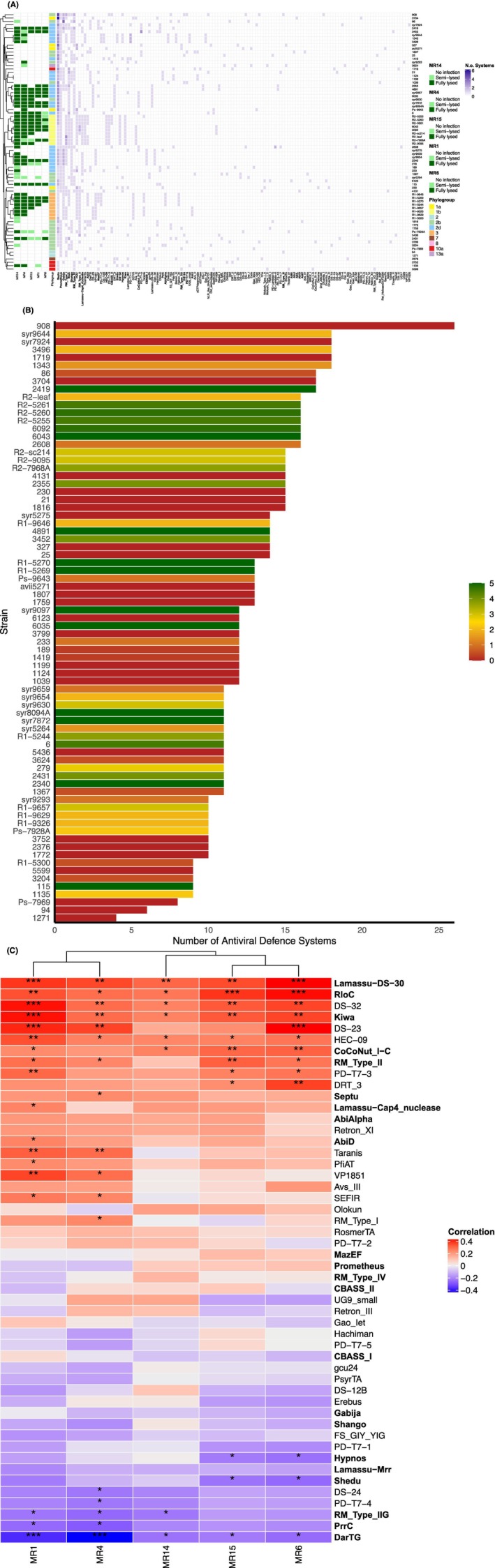
Relationship between phage infectivity and bacteria defence genes in 76 *Pseudomonas* strains. (A) Clustered frequency heatmap comparing 76 *Ps* strain defence profiles with the host range of 5 MR phages. Shades of purple indicate the presence of defence systems subtypes, whilst white indicates their absence. Darker shades of purple indicate that multiple copies of this defence system gene were present. A secondary heatmap accompanying this indicated the phage host range. Dark green indicated that strains were fully lysed by the phage, light green indicated they were semi‐lysed and white indicated they were resistant to lysis or infection. Strain names were accompanied by metadata concerning their phylogroup within the *Ps* complex. (B) Bar chart showing the total number of antiviral defence systems possessed by *Ps* strains, alongside susceptibility to phage lysis. Dark green bars indicated that strains were susceptible to all five phages, while dark red indicated they were resistant to lysis by all five MR phages. (C) Heatmap showing the Pearson correlation coefficient between phage infectivity and the presence of individual ADS subtypes. Darker shades of blue indicate a stronger negative relationship between phage resistance and ADS subtype presence, while darker shades of red indicate a stronger positive relationship between phage susceptibility and ADS subtype presence. Asterisks represent a significant relationship: Single asterisk, *p* < 0.05; double asterisk, *p* < 0.01, triple asterisk, *p* < 0.001. ADS discussed within the text are in bold.

Side‐by‐side comparison of ADS profiles and phage host range revealed a complex relationship between prokaryotic defence and susceptibility to phage infection (Figure 4A). Six strains in the phage‐susceptible supercluster centred around syr9097 (#2355, #4891, #6035, syr7872, syr8094A and syr9630) shared a similar defence profile. However, strain #1719, resistant to all five phages, had a similar defence profile to this subgroup, suggesting a more nuanced dynamic beyond the simple presence/absence of systems. Strains tentatively cluster by both phylogroup and susceptibility to lysis, but not by resistance. Three highly susceptible clusters aligned with phylogroups 1b, 2d and 3, yet some strains with similar profiles, like #1719 (2d‐like) and #1759 (1b‐like), were completely resistant. Identical defence profiles sometimes matched susceptibility (e.g., R1‐5270 and R1‐5269), but not always, for example, #279 and #2340 had identical profiles, yet #279 was less susceptible to MR1, MR6 and MR15. Small variations in defence genes may have large effects: #1343 (susceptible to all phages) closely resembled #2419 in defence profile, yet #2419 was fully resistant. To test if the total number of defence systems impacted resistance, we compared defence counts with resistance levels (Figure 4B). No clear relationship was found; strains like #908 (many systems) were as resistant as those with fewer (e.g., #94, #1271). This contrasts with other findings in *Pseudomonas* spp. and suggests that resistance is likely conferred by specific defence systems rather than cumulative effects.

Specific ADS subtypes were strongly associated with either resistance or susceptibility (Figure 4C). DarTG was significantly correlated with resistance to all five phages, while PrrC, Shedu, PD‐T7‐4, DS‐24, and Hypnos were linked to resistance against fewer (*p* < 0.05). Conversely, several ADS types—DS‐32, CoCoNut_1‐C, HEC‐09, Kiwa, Lamassu‐DS‐30 and RloC, were significantly correlated with susceptibility, with Lamassu‐DS‐30 and RloC being the most significant (*p* < 0.05). These correlations may indicate that some systems fail to confer protection, rather than actively promoting susceptibility.

### 3.4 LPS‐Associated Genes gpt, gst1 and lpk Were Highly Conserved but Only gpt Appeared to Influence Phage Infectivity

Three LPS biosynthesis genes, including *gst1*, *lpk* and *gpt* from the strain syr9097, identified as MR receptors in Rabiey et al. (2024), were used as references to retrieve homologues from other *Pseudomonas* genomes. A multiple sequence alignment of 250 *Pseudomonas* strains was performed using these genes (Figure S1A–) (Rabiey et al. 2024). Of the 250 strains, 94 lacked a *gst1* gene homologous to that of syr9097, all belonged to phylogroup 10a or fell outside the *Ps* complex. Ten strains had identical *gst1, gpt, and lpk* sequences to syr9097 (e.g., #2355, #4891, syr8094A), and an additional 10 shared only *gpt*. Strains with all three identical genes were lysed by all five phages, except for #2355 (resistant to MR15) and syr9630 (resistant to MR14 and MR15). Notably, #2355 and #4891 had nearly identical ADS profiles, differing only by an extra *RloC* gene in #4891. Other strains with duplicate *RloC* genes (e.g., #6, #21, #2341) showed varied MR15 resistance, suggesting *RloC* alone may not explain the phenotype.

**FIGURE 5:**
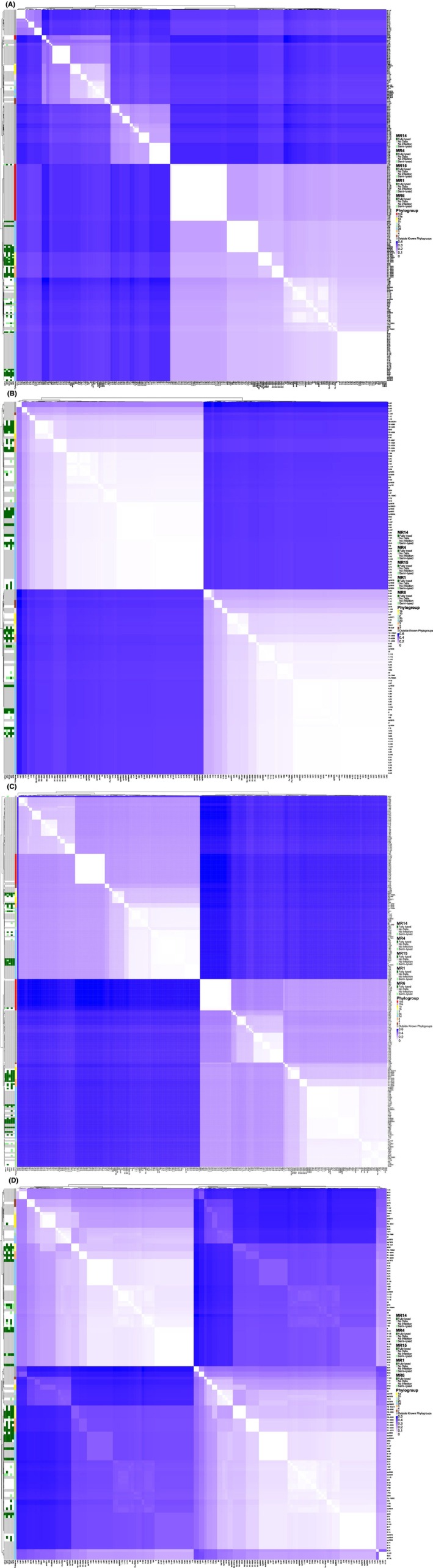
Pairwise correlation of the phylogenetic distances LPS receptor genes between 250 *Pseudomonas* strains. Darker shades of blue indicate a greater phylogenetic distance between strains, and therefore a greater difference in their gene sequences. Annotations include the phylogroup within the *Ps* complex to which strains belong to, alongside the host range of five MR phages. Dark green indicated that strains were fully lysed by the phage, light green indicated they were semi‐lysed and white indicated they were resistant to lysis or infection. Strains were clustered according to similarities in their gene sequences. Heatmaps each indicated a different LPS receptor gene: (A) *gpt* (glucose‐1‐phosphate thymidylyltransferase, BKC06_005130), (B) *gst1* (glycosyltransferase family 1, BKC06_002880), (C) *lpk* (lipopolysaccharide kinase, BKC06_002845) and (D) combined mean phylogenetic distance of *gpt*, *gst1* and *lpk* genes.

Phylogenetic analysis grouped strains by similarity in *gst1, gpt, and lpk*, which clustered by phylogroup and sub‐phylogroup (Figure 5A–C), indicating conservation within lineages. While *gst1* and *lpk* showed no clear link to phage susceptibility, *gpt* was strongly associated with host range. When combining phylogenetic distances of all three genes (Figure 5D), no overall relationship with host range was observed, supporting the idea that *gpt* alone influences phage infectivity.

## 4 Discussion

This study emphasises the necessity of integrating bacterial ADS profiling and receptor analysis into phage host‐range assessments. Our data reveal a multifaceted interplay between ADS, LPS‐related receptor genes (especially *gpt*) and phage infectivity. Although genomic features like ADS and receptor variants help predict phage susceptibility, they cannot fully substitute empirical assays. Indeed, plaque and killing‐curve assays remain indispensable for determining phage infectivity given the multifactorial nature of resistance. Even when candidate phages are selected based on genomic analyses—such as targeting receptors or accounting for common ADS—these empirical assays are essential to confirm actual infectivity and host range.

Consistent with Hulin et al. (2023), who observed conservation of virulence factors across phylogroups in *P. syringae* (Hulin, Armitage, et al. 2018; Costa et al. 2024), we found that both defence profiles and receptor genes are phylogenetically conserved. Specifically, strains with identical or near‐identical *gpt* sequences, central to the syr9097 supercluster, shared susceptibility to MR phages. This aligns with recent findings implicating LPS biosynthetic genes as primary receptors for MR phages (Rabiey et al. 2024). However, *gst1* and *lpk* variants showed weaker or no discernible correlation with host range, reinforcing the dominant role of *gpt* in MR phage adsorption. Despite identical *gpt* sequences, ADS variation significantly influenced susceptibility. For example, phylogroup 3 strains with syr9097‐like *gpt* but divergent ADS profiles displayed different lysis patterns, suggesting even minor ADS differences can alter infectivity. Notably, PrrC correlated significantly with resistance to MR4 and MR14, indicating specific subtypes can play outsized roles. Across the 76‐strain panel, DarTG, a toxin–antitoxin ADS, was the only subtype significantly associated with resistance to all five MR phages. This corresponds well with recent studies demonstrating DarTG's broad‐spectrum anti‐phage activity by ADP‐ribosylating viral DNA (LeRoux et al. 2022). Conversely, Lamassu‐DS‐30, RloC, and CoCoNut_1‐C correlated with increased susceptibility. These ADS are believed to function through abortive infection, inducing bacterial death upon activation (Macdonald et al. 2023). This raises a hypothesis that population declines in killing‐curve assays may result from ADS‐triggered suicide rather than phage replication. While abortive systems serve to contain phages in natural settings, they can produce misleading positive results in vitro if interpreted solely as indicators of phage activity.

These findings suggest that in clinical contexts, such as treating *P. aeruginosa* infections, understanding both receptor diversity and specific ADS profiles could improve the selection and effectiveness of personalised phage therapies. Our findings confirm observations in *P. aeruginosa* , where total ADS count correlated with phage resistance (Costa et al. 2024). However, unlike *P. aeruginosa* , our *P. syringae* strains did not show a phage‐resistance dependence on ADS quantity; instead, specific ADS types and receptors were decisive. This discrepancy likely reflects differences in receptor diversity: *P. aeruginosa* exhibits high O‐antigen heterogeneity, while our data suggest *P. syringae* defences may be more receptor‐focused, particularly on conserved LPS biosynthesis genes. Müller et al. (2024) also showed that the presence or absence of bacterial anti‐phage defence systems (e.g., CRISPR, RMs) had no significant impact on whether the phages could infect a host strain. Instead, differences in surface receptor compatibility were the primary determinant of host range, which confirms our finding as well (Müller et al. 2024).

However, expanding comparative analyses to a broader *Pseudomonas* range will help determine whether the patterns observed here are generalisable across the genus. Receptor conservation and ADS composition may differ substantially in other species or environmental contexts, potentially altering phage–host dynamics. Also, gene presence does not guarantee activity; therefore, functional validation of candidate ADS can confirm their function. Moreover, our killing‐curve assays were limited in number; further replication across more strain–phage combinations will be essential and additional receptor mapping approaches could provide more mechanistic insight into how ADS–receptor interactions shape infection outcomes.

Overall, our study supports a model in which phage susceptibility is primarily determined by key bacterial receptors, in combination with the presence or absence of specific ADS subtypes. Integrating these genomic features with experimental host range validation can enable the rational design of phage cocktails, substantially enhancing their effectiveness in agricultural biocontrol and potentially in clinical settings.

## Author Contributions

**Kieran Cooney‐Nutley:** methodology, investigation, validation, formal analysis, data curation, visualization, writing – original draft, writing – review and editing, conceptualization. **Sneha Chakravorty:** writing – review and editing, methodology, investigation. **Imogen Nix:** investigation, methodology, writing – review and editing. **Ziyue Zeng:** writing – review and editing, methodology. **Shannon F. Greer:** conceptualization, writing – review and editing, supervision, methodology, visualization, validation. **Mojgan Rabiey:** conceptualization, investigation, funding acquisition, writing – original draft, writing – review and editing, visualization, validation, methodology, formal analysis, project administration, resources, supervision, data curation.

## Conflicts of Interest

The authors declare no conflicts of interest.

## Supporting information


**Figure S1:** Phylogenetic trees of phage receptor genes in 250 *Pseudomonas* strains, with a focus on the *Ps* complex. Three LPS genes were used from *Pseudomonas syringae* pv, *syringae* strain 9097 (accession number: CP026568) to align with homologues located in other bacterial genomes. These were: (A) *gpt* (glucose‐1‐phosphate thymidylyltransferase, BKC06_005130), (B) *gst1* (glycosyltransferase family 1, BKC06_002880) and (C) *lpk* (lipopolysaccharide kinase, BKC06_002845).

**Table S1:** Information describing *Pseudomonas* strains analysed in this study.

**Table S2:** Generalised linear model (Type III ANOVA) SPSS output analysing the factors that may impact Pseudomonas strain antiviral arsenal size.

## Data Availability

The datasets supporting the conclusions of this article are included within the article and its Supporting Information.
