# Supplementary figures and images for: Understanding Bacterial Antiviral Defence Systems and Phage Receptors to Better Inform Rational Phage Cocktail Design to Treat Bacterial Canker
Source: Microb Biotechnol. 2025 Sep 19;18(9):e70232. doi: 10.1111/1751-7915.70232 (PMC12447246; doi:10.1111/1751-7915.70232)

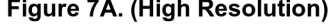

Supplement: Supplementary file 1 — Figure S1: Phylogenetic trees of phage receptor genes in 250 Pseudomonas strains, with a focus on the Ps complex. Three LPS genes were used from Pseudomonas syringae pv, syringae strain 9097 (accession number: CP026568) to align with homologues located in other bacterial genomes. These were: (A) gpt (glucose‐1‐phosphate thymidylyltransferase, BKC06_005130), (B) gst1 (glycosyltransferase family 1, BKC06_002880) and (C) lpk (lipopolysaccharide kinase, BKC06_002845). [file MBT2-18-e70232-s002.docx › mbt270232-sup-0001-FigureS1@Figure_S1A.pdf]

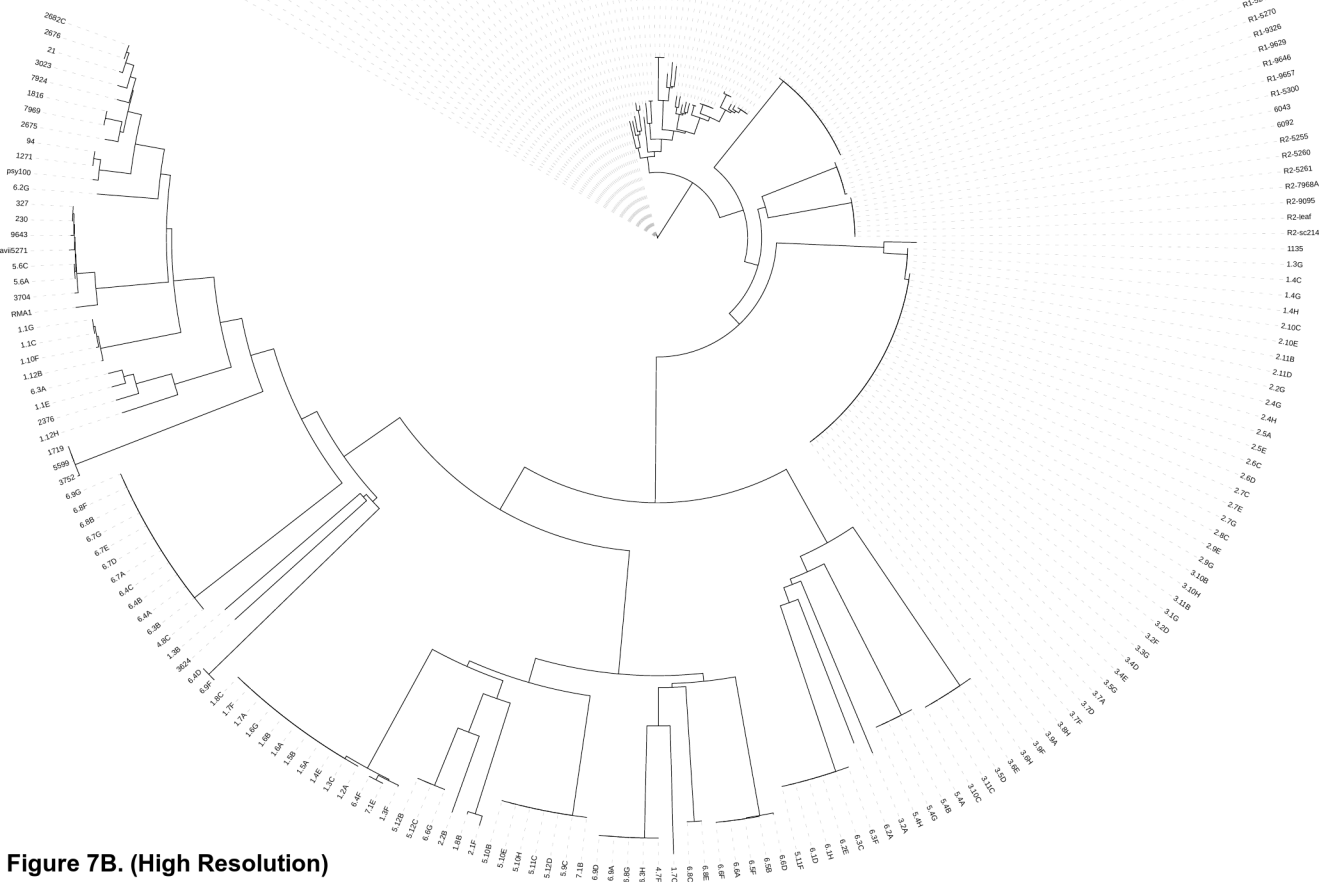

Supplement: Supplementary file 1 — Figure S1: Phylogenetic trees of phage receptor genes in 250 Pseudomonas strains, with a focus on the Ps complex. Three LPS genes were used from Pseudomonas syringae pv, syringae strain 9097 (accession number: CP026568) to align with homologues located in other bacterial genomes. These were: (A) gpt (glucose‐1‐phosphate thymidylyltransferase, BKC06_005130), (B) gst1 (glycosyltransferase family 1, BKC06_002880) and (C) lpk (lipopolysaccharide kinase, BKC06_002845). [file MBT2-18-e70232-s002.docx › mbt270232-sup-0002-FigureS1@Figure_S1B.pdf]
